# Supplementary material for: Prevalence of Worry-Induced Sleep Disturbance and Associated Factors among a National Sample of In-School Adolescents in Lebanon
Source: Behav Sci (Basel). 2020 Sep 27;10(10):148. doi: 10.3390/bs10100148 (PMC7600147; doi:10.3390/bs10100148)
Supplement: Supplementary file 1 [file behavsci-10-00148-s001.pdf]

Supplementary file 1: Variable description

Variables	Question	Response options (coding scheme)
Anxiety	"During the past 12 months, how often have you been so worried about something that you could not sleep at night?"	"1=never to 5=always (coded 1–3=0 and 4–5=1)"
Age	"How old are you?"	"11 years old or younger to 18 years old or older"
Sex	"What is your sex?"	"Male, Female"
	Psychosocial distress	
No close friends	"How many close friends do you have?"	"1 = 0 to 4 = 3 or more (coded 1+=0, 0=1)"
Loneliness	"During the past 12 months, how often have you felt lonely?"	"1=never to 5=always (coded 1–3=0 and 4–5=1)"
Bullied	"During the past 30 days, on how many days were you bullied?"	"1=0 days to 7=All 30 days"
Physically attacked	"During the past 12 months, how many times were you physically attacked?"	"1=0 times to 8=12 or more times"
Physical fights	"During the past 12 months, how many times were you in a physical fight?"	"1=0 times to 8=12 or more times"
	Social-environmental factos	
Hunger	"During the past 30 days, how often did you go hungry because there was not enough food in your home?"	"1 = never to 5 = always (coded 1-3=0 and 4-5=1)"
Peer support	"During the past 30 days, how often were most of the students in your school kind and helpful?"	"1=never to 5=always (coded 1-2=1, 3–5=0)"
Parental supervision	"During the past 30 days, how often did your parents or guardians check to see if your homework was done?"	"1=never to 5=always (coded 1=1 and 2–5=0)"
Parental emotional neglect	"During the past 30 days, how often did your parents or guardians understand your problems and worries?"	"1=never to 5=always (coded 1=1 and 2-5=0)"
	"During the past 30 days, how often did your parents or guardians really know what you were doing with your free time?	"1=never to 5=always (coded 1-2=1 and 3-5=0)"
Parental disrespect for privacy	"During the past 30 days, how often did your parents or guardians go through your things without your approval?"	"1=never to 5=always (coded 1–3=0 and 4–5=1)"
Passive smoking	"During the past 7 days, on how many days have people smoked in your presence?"	"1=0 days to 5=all 7 days"
School truancy	"During the past 30 days, on how many days did you miss classes or school without permission?"	"1=0 days to 5= 10 or more days"
	Health risk behaviours	
Drunk	"During your life, how many times did you drink so much alcohol that you were really drunk?"	"1=0 times to 4=0 or more times (coded 1=0 and 2-4=1)"
Current tobacco use	"During the past 30 days, on how many days did you smoke cigarettes/use any tobacco products other than cigarettes, such as a narghile or waterpipe?"	"1=0 days to 7=All 30 days (coded 1=0 and 2-7=1)"
Cannabis use	"During your life, how many times have you used marijuana (also called hash or hashish)?"	1=0 times to 5=20 or more times) (coded 1=0 and 2-4=1)"
Leisure-time sedentary behavior	"How much time do you spend during a typical or usual day sitting and watching television, playing computer games, talking with friends, or doing other sitting activities, such as playing with a PC or video games, playing on an Ipad or other tablet, or chatting with your friends on the phone?"	"1=Less than 1 hour per day… 3= 3 to 4 hours per day …6=8 or more hours a day"
Soft drink intake	"During the past 30 days, how many times per day did you usually drink carbonated soft drinks, such as Pepsi, Coca Cola, Fanta, or 7-Up? (Do not include diet soft drinks.)"	"1=0 times to 7=5 or more times (coded 1-2=0 and 3-7, 2 or more times)"
Fast food consumption	"During the past 7 days, on how many days did you eat food from a fast food restaurant, such as hamburger, shawarma, falafel, pizza, or mnakish?"	"1=0 days to 8=7 days (coded 1-2=0 and 3-6=1, 2 or more days)"
Injury	"During the past 12 months, how many times were you seriously injured?"	"1=0 times to 8=12 or more times (coded 1=0 and 2–8=1)"
